# Supplementary material for: Whole Exome Sequencing for the Identification of Mutations in Bone Marrow CD34+Cells in Hodgkin Lymphoma
Source: Curr Issues Mol Biol. 2025 Oct 23;47(11):880. doi: 10.3390/cimb47110880 (PMC12651673; doi:10.3390/cimb47110880)
Supplement: Supplementary file 1 [file cimb-47-00880-s001.zip › cimb-3908112-supplementary.pdf]

Supplementary Table S1. Genotype distribution of gene variants in cHL patients.

Gene Name	dbSNP ID	Type of Variant	Transcription	Locus	Nucleotide Change	Amino Acid Change	Variant Allele Frequency (%)	Scores of SIFT/PolyPhen/LRT/MutationTaster/MutationAssessor/FATHMM	SIFT/PolyPhen/LRT/MutationTaster/MutationAssessor/FATHMM	ExAC/1000 Genome (Frequency)	ClinVar	Alpha Fold Database (Model Confidence)
**Lymphoma- and/or leukemia-related gene**												
MUC4	Novel	Non-frameshift deletion	NM_004532.5	Exon 1	c.3228_3371DelGGTGGTGTGACCTGTGGATACTGAGGAAGTGTCGGTGACAGGAAGAGAGGTGGCGTGACCTGTGGATGCTGAGGAAGTGTCGGTGACAGGAAGAGGGGTGGTGTGACCTGTGGATACTGAGGAAGTGTCGGTGACAGGAAGAGA	p.P1076_P1124Del	33.33	./././././.	./././././.	0.005/0	Pathogenic	High
	Novel	Non-frameshift insertion	NM_004532.5	Exon 1	c.4826InsTGGTGACAGGAAGAGGGGTGGCGTGACCTGTGGATGCTGAGGAAGGGC	p.P1609Ins	23.33	./././././.	./././././.	0.0008/0	Pathogenic	Very high
	rs748236754	Frameshift insertion	NM_004532.5	Exon 1	c.4922InsGTGGATACTGAGGAAGTGTC	p.P1641Ins	26.67	./././././.	./././././.	0.0023/0	Pathogenic	Very high
	Novel	Non-frameshift deletion	NM_004532.5	Exon 1	c.9832_9927DelCTGAGGAAGGGCTGGTGACATGAAGAGGGGTGGCGTGACCTGTGGATGCTGAGGAAGCGTCGGTGACAAGAAGAGGAGTGGCGTGACCTGTGGATA	p.Y3278_Q3209Del	66.67	./././././.	./././././.	0.0005/0	Pathogenic	Unknown

	Novel	Non-frameshift deletion	NM_004532.5	Exon 1	c.12363_12410 DelGGTGACAGGAAGA-GAGGTGGTGTGACCTGAG-GATGCTGAG-GAAGGGAT	p.P4121_P4137Del	16.67	./././././.	./././././.	0.000065/0	Pathogenic	Unknown
	rs748705487	Non-frameshift deletion	NM_004532.5	Exon 1	c.12414_12461 DelGCTGGTGACAGGAA-GAGGGGTGCCGTGACCTGTG-GACACTGAG-GAAGC	p.T4138_A4154Del	20	./././././.	./././././.	0.0062/0	Pathogenic	Unknown
**Cancer-associated gene**												
ABCA4	rs142253670	Missense	NM_000350.3	Exon 42	c.5881 G > A	p.G1961R	3.33	0.001/1/0.843/1/0.3/0.937	D/D/D/D/N/D	0.000091/0.0002	Likely pathogenic	High
ACADSB	rs553730391	Missense	NM_001609.4	Exon 5	c.655 G > A	p.V219M	3.33	0/1/0.843/1/3.535/0.976	D/D/D/D/H/D	0.000056/0.0002	Pathogenic	Very high
ADIPOQ	rs121917815	Missense	NM_004797.4	Exon 3	c.334 C > T	p.R112C	3.33	0/1/0.537/1/3.435/0.86	D/D/D/A/M/D	0.000012/0	Pathogenic	High
AGXT	rs34116584	Missense	NM_000030.3	Exon 1	c.32 C > G	p.P11R	3.33	0.001/1/0.843/1/3.205/0.9	D/D/D/D/M/D	0.0002/0	Pathogenic	Very high
GCGR	rs1219737977	Missense	NM_000160.5	Exon 4	c.187 G > A	p.D63N	3.33	0.025/0.999/0.629/1/3.5/0.747	D/D/D/D/M/T	0.000035/0	Pathogenic	Very high
MTSS2	rs531163149	Missense	NM_138383.3	Exon 15	c.1790C > T	p.T597M	13.33	0.001/1/0.843/1/2.56/0.376	D/D/D/D/M/T	0.0001/0.000599	Likely pathogenic	Low
STIL	rs369348360	Missense	NM_001048166.1	Exon 12	c.1226G > C	p.S409T	3.33	0.077/0.992/0.629/0.91/2.785/0.502	T/D/D/D/M/T	0.00002/0	Likely pathogenic	Low
TH	rs1264884607	Missense	NM_000360.4	Exon 11	c.1189G > A	p.G397R	3.33	0/1/0.843/1/3.335/0.997	D/D/D/D/M/D	0.00002/0	Likely pathogenic	High
AFAP1L1	rs140123570	Missense	NM_152406.4	Exon 15	c.1785 G > C	p.Q595H	10	0.118/0.996/0.629/0.993/2.095/0.916	T/D/D/D/M/D	0.0000795/0.0002	USV	Low
FBN3	rs540022951	Missense	NM_032447.5	Exon 53	c.6550 A > G	p.T2184A	10	0.152/0.941/0.843/1/2.595/0.928	T/P/D/D/M/D	0.000599/0.0000804	USV	Unknown
TTLL5	rs375900619	Missense	NM_015072.5	Exon 26	c.2966 G > A	p.R989H	10	0.152/0.941/0.331/1/2.595/0.928	D/D/D/D/M/T	0.00019968/0.0000478	USV	Low

SIFT, “D” meaning damaging, score <0.05, “T” meaning tolerated, score ≥0.05; PolyPhen2, “D” meaning damaging, 0.957 ≤ score ≤ 1, “P” meaning possibly damaging, 0.453 ≤ score ≤ 0.956, “B” meaning benign, 0 ≤ score ≤ 0.452; LRT, “D” meaning deleterious, “U” meaning unknown; MutationTaster, “A” and “D” represent as disease causing automatic and deleterious, 0.84 ≤ score ≤ 1; MutationAssessor, “H” meaning high impact, score > 3.5, “M” meaning medium impact, 1.9 ≤ score ≤ 3.5, “N” meaning neutral impact, score ≤ 0.8; FATHMM, “D” meaning damaging and “T” meaning tolerated, score >0.5, “N” meaning neutral, score <0.5; “0” in frequency or “.” means didn’t find in the database.

Supplementary Table S2. Association between CNN2 and MUC4 expression levels and clinical parameters in cHL patients.

Parameters	Normal Range	*CNN2* Expression Level			*MUC4* Expression Level		
		**High (n = 42)**	**Low (n = 18)**	***p* Value**	**High (n = 34)**	**Low (n = 26)**	***p* Value**
Age (years)		36.9 (16–76)	33.95 (17–76)	0.532	34.74 (16–76)	36.26 (16–70)	0.734
Sex, Female (n, %)		19 (45.24)	7 (38.89)	0.547	15 (44.11)	10 (38.46)	0.645
Urea (mmol/l)	3.3–6.6	4.56 ± 1.16	4.87 ± 1.76	0.415	4.66 ± 1.14	4.72 ± 1.72	0.648
Glucose (mmol/l)	3.9–5.6	6.2 ± 7.44	4.77 ± 1.09	0.815	6.23 ± 8.43	5.17 ± 1.73	0.788
Creatinine (μmol/l)	50–110	73.85 ± 17.26	70.48 ± 15.3	0.924	70.94 ± 17.4	74.46 ± 16.67	0.377
Uric acid (μmol/l)	<420	323.28 ± 73.11	318.36 ± 99.42	0.472	327.73 ± 69.29	324.73 ± 99.32	0.894
Total bilirubin (μmol/l)	0–21	13.09 ± 23.05	16.46 ± 31.64	0,.54	23.25 ± 25.85	10.5 ± 7.19	0.713
Direct bilirubin (μmol/l)	0–7	5.66 ± 15.62	7.01 ± 20.4	0.791	6.14 ± 18.5	3.01 ± 2.72	0.939
Indirect bilirubin (μmol/l)	1–17	9.33 ± 11.87	12.09 ± 15.52	0.632	11.3 ± 14.58	6.88 ± 2.54	0.219
Total protein (g/l)	60–80	79.18 ± 8.95	77.45 ± 7.46	0.442	79.47 ± 7.81	79.71 ± 8.86	1
Albumin (g/l)	35–52	38.99 ± 5.08	38.34 ± 5.8	0.96	39.99 ± 4.71	37.96 ± 6	0.41
Globulin (g/l)	20–35	41.02 ± 10.25	40 ± 8.96	0.429	39.68 ± 8.48	42.88 ± 10.79	0.156
Ferritin (ng/mL)	10–300	667.69 ± 751.4	975.1 ± 703.3	0.562	630.89 ± 718.02	849.34 ± 743.43	1
AST (GOT) (U/l)	5–40	25.9 ± 16.6	24.82 ± 14.09	0.639	24.89 ± 14.78	25.25 ± 15.96	0.617
ALT (GPT) (U/l)	7–55	35.47 ± 36.87	33.39 ± 30.38	0.411	32.6 ± 32.74	39.55 ± 38.76	0.893
LDH (U/l)	0–247	350.96 ± 244.76	370.64 ± 186.26	**0.048**	293.58 ± 146.08	384.27 ± 262.84	0.169
β2 microglobulin (mg/L)	0.8–2.4	2.83 ± 2.13	2.47 ± 1.3	0.195	2.53 ± 1.59	2.9 ± 2.22	0.252
Erythrocytes (10*12 cells/L)	4.2–5.9	4.6 ± 0.74	4.4 ± 0.93	0.12	4.76 ± 0.69	4.4 ± 0.88	0.167
Hemoglobin (g/L)	130–180	124.42 ± 24.75	122 ± 23.11	0.188	128.12 ± 24.65	120.62 ± 23.49	0.131
Hematocrit (%)	42–52	39 ± 6	37 ± 6	**0.039**	40 ± 5	38 ± 7	0.115
WBC count (×10*9/L)	5–14.5	11.74 ± 5.03	11.85 ± 6.78	0.528	11.55 ± 5.6	12.9 ± 5.46	0.515
Neutrophil count (×10*9/L)	1.6–7.5	8.27 ± 4.35	8.97 ± 6.13	0.211	7.8 ± 4.96	9.97 ± 4.95	0.157
Lymphocyte count (×10*9/L)	0.9–3.4	1.96 ± 2.3	1.64 ± 0.95	0.744	2.23 ± 2.59	1.56 ± 0.65	0.328
Monocyte count (×10*9/L)	0–1.2	0.73 ± 0.35	0.79 ± 0.43	0.632	0.75 ± 0.36	0.77 ± 0.32	0.784
Eosinophil count (×10*9/L)	0–0.8	0.27 ± 0.22	0.3 ± 0.29	0.706	0.31 ± 0.26	0.24 ± 0.22	0.437
Basophil count (×10*9/L)	0–0.3	0.074 ± 0.08	0.08 ± 0.07	0.13	0.07 ± 0.07	0.09 ± 0.1	1
Platelet count (× 10*9/L)	150–400	341.19 ± 115.44	355.14 ± 119.54	0.562	337.64 ± 91.37	365.11 ± 139.7	0.199
NLR		5.66 ± 3.19	6.8 ± 5.66	0.298	4.95 ± 3.48	7.17 ± 4.54	**0.037**

ALT, alanine aminotransferase; AST, aspartate aminotransferase; LDH, lactate dehydrogenase; NLR, neutrophil-to-lymphocyte ratio; and WBC, white blood cells. Level of significance $p < 0.05$. The bold type indicates statistical significance.
